# Oxidative Stress and Skin Diseases: The Role of Lipid Peroxidation

**DOI:** 10.3390/antiox14050555

**Published:** 2025-05-07

**Authors:** Federica Li Pomi, Luca Gammeri, Francesco Borgia, Mario Di Gioacchino, Sebastiano Gangemi

**Affiliations:** 1 Department of Precision Medicine in Medical, Surgical and Critical Care (Me.Pre.C.C.), University of Palermo, 90127 Palermo, Italy; federicalipomi@hotmail.it; 2 Department of Biomedical and Dental Science and Morphofunctional Imaging, University of Messina, 98125 Messina, Italy; lucagammeri@outlook.com; 3 Section of Dermatology, Department of Clinical and Experimental Medicine, University of Messina, 98125 Messina, Italy; fborgia@unime.it; 4 Institute for Clinical Immunotherapy and Advanced Biological Treatments, 65100 Pescara, Italy; 5 Department of Clinical and Experimental Medicine, School and Operative Unit of Allergy and Clinical Immunology, University of Messina, 98125 Messina, Italy; sebastiano.gangemi@unime.it

**Keywords:** lipid peroxidation, oxidative stress, 4-hydroxynonenal, malondialdehyde, skin, melanoma, ferroptosis, vitiligo, psoriasis, atopic dermatitis

## Abstract

Lipid peroxidation (LPO) is a biochemical process through which lipids are subjected to a peroxidation reaction in the presence of free radicals. The process can cause alterations in biological membranes and the formation of substances harmful to the body that can form aggregates with proteins and nucleic acids. Malondialdehyde (MDA) and 4-hydroxynonenal (4-HNE) are the main products of LPO. These compounds have cytotoxic and genotoxic properties and contribute to the pathogenesis of various diseases. This research focuses on the correlation between LPO and skin diseases. For some skin diseases, such as psoriasis, vitiligo, and alopecia, LPO products have been shown to have a clear role in the pathogenesis of the disease. Lipid aldehydic products like MDA and 4-HNE can enhance inflammation by stimulating pro-inflammatory genes and producing cytokines. Furthermore, these products can stimulate cell death and increase oxidative stress. For other diseases (atopic dermatitis, urticaria, pemphigus, and melanoma), the role of LPO is unclear, even if the levels of LPO biomarkers are elevated in proportion to the severity of the disease. LPO can also be exploited to counteract the proliferation of neoplastic cells. Therefore, enhancing LPO would play an adjuvant role in the therapy of neoplastic diseases such as melanoma. In particular, the therapeutic implication resulting from the role of LPO products in the cytotoxicity induced by photodynamic therapy used for the adjuvant treatment of melanoma could be of interest in the future.

## 1. Introduction

Lipid peroxidation (LPO) is a process by which lipids are subjected to the peroxidation process in the presence of free radicals and reactive oxygen species (ROS). The correlation between oxidative stress, its products, and inflammatory diseases has been widely demonstrated [1]. Lipids are essential macromolecules for the integrity of biomembranes in animal and plant organisms, so lipid peroxidation is harmful to cells. Lipid changes in the phospholipid bilayer result in alterations in membrane fluidity, ion channel activity, and membrane permeability [2]. Peroxidation byproducts can also exacerbate ROS production and bind to DNA and proteins, damaging them. Lipid peroxides can be produced by two specific pathways: enzymatic and non-enzymatic pathways. Cyclooxygenases, cytochrome p450, or lipoxygenases regulate the enzymatic pathway [3]. The enzymatic action of cyclooxygenases produces endoperoxides as intermediate products, which are important in inflammatory processes [4]. Lipoxygenases and non-enzymatic reactions instead lead to the production of the most harmful products of LPO, namely hydroperoxides. In the non-enzymatic pathway, the imbalance between redox systems triggers the process. The disruption of this balance can be linked to the production of excessive quantities of oxidizing molecules or to the inefficiency of antioxidant systems. ROS induces the LPO chain reaction, which activates different processes whose final result is cell death. This process can induce cell apoptosis by regulating the nuclear factor kappa-light-chain-enhancer of activated B cells (NF-κB) pathway, mitogen-activated protein kinases (MAPKs), or protein kinase C (PKC) and autophagy through the promotion of the AMPK/mTORC and JNK-Bcl-2/Beclin 1 pathways [5]. Lipid peroxides are also essential in ferroptosis, a non-apoptotic mechanism of cell death characterized and driven by exaggerated membrane lipid peroxidation. This process leads to altered ionic fluxes and plasma membrane permeabilization [6]. The lipids most affected by the process are long-chain polyunsaturated fatty acids (PUFAs) containing many carbon–carbon double bonds. The initiation of the process is catalyzed by the intracellular iron pool, which reacts with hydrogen peroxide and superoxide to produce oxygen-centered radicals. This type of reaction is called “Fenton chemistry” [7]. Oxidizing agents strip allylic hydrogen from the carbon that bridges the double bonds, forming unstable lipid radicals (L・) that rapidly react with an oxygen molecule to generate lipid peroxide radicals (LOO・). The latter strips another hydrogen from a different lipid molecule, forming more stable compounds called lipid hydroperoxides (LOOH) [8].

The main end products of the lipid peroxidation process are aldehydes, particularly malondialdehyde (MDA), 4-hydroxynonenal (4-HNE), propanal, and hexanal [8,9].

MDA is an end product of LPO generated by the decomposition of arachidonic acid and PUFA. It may have a dual biological function that is dose dependent. This molecule may act as a signaling messenger and regulate gene expression. On the other hand, non-enzymatic processes related to increased oxidative stress (OS) lead to the excessive accumulation of MDA, which has a high capacity to react with multiple biomolecules such as proteins and DNA [8]. MDA is widely used as the primary biomarker of OS since it is easily measurable in biological fluids (blood and urine) and exhaled air [10]. This end product is the most mutagenic LPO product [11]. It is known that high levels of MDA are correlated with a high inflammatory state, and to demonstrate this, MDA levels can be evaluated, for example, for monitoring asthmatic patients [12]. 4-HNE is the most important and dangerous lipid peroxidation product due to its cytotoxic and genotoxic effects. When present in high concentrations, this compound promotes cell damage and the activation of cell death mechanisms. 4-HNE also has a signaling function and is involved in the regulation of several transcription factors sensitive to OS, such as nuclear factor erythroid 2-related factor 2 (Nrf2), activating protein-1 (AP-1), NF-κB, and peroxisome proliferator-activated receptors (PPARs) [8]. OS plays an important role in many pathologies, so there is an increasing interest in OS biomarkers. OS contributes to the genesis and the maintenance of chronic inflammatory processes, as well as neurodegenerative processes such as Alzheimer’s disease [13] and Parkinson’s disease [14]. Elevated levels of oxidative biomarkers have also been found in the context of cardio-metabolic disorders [15,16]. Furthermore, increased values of OS markers and, in particular, of LPO, have been found both in the context of benign proliferative processes, such as benign prostatic hypertrophy [17], and in the course of lymphoproliferative processes [18] or in the development of cancer [19].

LPO seems to play a dual role in the skin, contributing both to homeostasis and the maintenance of physiological mechanisms, as well as to the onset of various diseases. As the primary interface between the body and the environment, the skin is continuously exposed to OS from endogenous metabolic processes and exogenous and environmental factors, including ultraviolet (UV) radiation and pollution [20]. The external exposome encompasses all environmental exposures, including pollutants, diet, and lifestyle, which contribute to OS by enhancing ROS production and impairing antioxidant defenses [21].

Urban air pollutants, including particulate matter (PM), ozone (O₃), and nitrogen dioxide (NO₂), have been shown to penetrate the stratum corneum and directly induce OS by activating toll-like receptors (TLRs) and the aryl hydrocarbon receptor (AhR) pathway [22]. These agents, while triggering ROS production, also impair endogenous antioxidant defenses, thereby enhancing lipid oxidation. Also, volatile organic compounds (VOCs) and certain preservatives in cosmetic products can act as indoor pro-oxidant cutaneous stressors, further enhancing the peroxidation cascade in keratinocytes [23,24]. Moreover, cigarette smoke and industrial emissions, rich in polycyclic aromatic hydrocarbons and transition metals, further contribute to the oxidative burden by catalyzing redox reactions on the skin surface [25]. Furthermore, UV radiation—particularly UVA—interacts with skin chromophores to generate ROS, thus triggering the peroxidation of epidermal lipids and exacerbating tissue damage.

This leads to the formation of lipid radicals and LOOH, which mainly decompose into MDA and 4-HNE, causing cytotoxic, genotoxic, and pro-inflammatory effects [8].

In the skin, these reactive aldehydes act as secondary messengers in redox-sensitive pathways, including NF-κB and Nrf2, thus influencing gene expression, protein modifications, and enzymatic activity, which are essential for maintaining epidermal integrity and function. Additionally, LPO is involved in barrier homeostasis by modulating the composition and organization of epidermal lipids, including ceramides and free fatty acids, thereby contributing to stratum corneum permeability and protection against environmental insults [23]. In response to physiological ROS levels, LPO also plays a role in adaptive responses to OS, activating enzymatic and non-enzymatic antioxidant defense mechanisms, including superoxide dismutase (SOD), catalase, glutathione peroxidase (GSH-Px), α-tocopherol, and β-carotene, which prevent excessive lipid oxidation, thereby preserving cellular function and structural stability [26].

Under normal conditions, this process is tightly regulated to prevent excessive oxidative damage, ensuring a balance between redox signaling and structural integrity within the skin microenvironment. However, when antioxidant defenses are overwhelmed, as occurs in pathological conditions, LPO products accumulate, triggering inflammatory cascades and oxidative damage. This imbalance is particularly relevant in both inflammatory and autoimmune skin disorders, where OS enhances disease onset and progression by altering lipid metabolism and impairing normal cellular responses [26,27]. Furthermore, LPO-derived metabolites are implicated in immune modulation, influencing the release of cytokines and mediators involved in skin inflammation [28].

Moving from these premises, this article explores the mechanisms by which LPO influences cutaneous diseases, shedding light on its role in pathogenesis and potential therapeutic interventions.

## 2. Materials and Methods

We performed a literature search using the PubMed database and the keywords “lipid peroxidation” and “skin”. We identified articles regarding both skin-inflammatory and non-inflammatory diseases. We excluded previous reviews and systematic reviews. Only English-language articles were included. We included all valuable articles published in the time frame between 1980 and March 2025.

## 3. Results

### 3.1. Psoriasis

Psoriasis is a chronic, immune-mediated inflammatory skin disorder characterized by erythematous-scaly plaques caused by keratinocyte hyperproliferation. It results from dysregulated interactions between the innate and adaptive immune systems, primarily involving the T helper (Th)1, Th17, and Th22 pathways, with the consequent release of interleukin (IL)-17, IL-23, and tumor necrosis factor α (TNF-α). Genetic predisposition and external triggers, including excessive OS, contribute to disease pathogenesis [29,30].

Moving to OS, evidence highlights that LPO contributes to the chronic inflammation and tissue damage characteristics of psoriasis pathophysiology.

Emerging evidence suggests that LPO is not merely a downstream consequence of psoriatic inflammation but may also play an active role in disease initiation and progression. Elevated OS can initiate LPO, which in turn modulates immune responses and keratinocyte behavior through mechanisms such as ferroptosis—a regulated form of cell death—and T-cell polarization [31,32]. Specifically, a significant correlation between lipid oxidation pathways and Th17/Th22 immune responses has been identified in psoriatic keratinocytes, suggesting that LPO may modulate immune signaling pathways involved in disease pathogenesis [33].

These findings support a bidirectional relationship, where LPO amplifies and is amplified by the chronic inflammatory state characteristic of psoriasis.

Consistent with this, increased levels of LPO markers—up to two-fold higher than in healthy controls—have been detected in both lesional skin and systemic circulation, correlating with disease severity, measured by the Psoriasis Area and Severity Index [34]. Thus, LPO not only exacerbates local skin inflammation but may also contribute to systemic oxidative damage in psoriatic patients [35], which correlates with the onset of other associated comorbidities, including cardiovascular diseases and psoriatic arthritis [36,37].

Among LPO products, 4-HNE is particularly relevant due to its ability to form covalent adducts with proteins, thereby disrupting cellular function and promoting inflammation. Due to its electrophilic nature, 4-HNE interacts with cysteine, histidine, and lysine residues in proteins, forming Schiff bases and Michael adducts that exhibit pro-oxidant, pro-inflammatory, and pro-apoptotic properties. These modifications contribute to psoriasis pathogenesis by altering intercellular signaling, immune responses, and keratinocyte proliferation [38]. Elevated levels of 4-HNE have been detected in several samples, including skin biopsies, various epidermal cell types, immune cells, and plasma [39,40,41,42].

4-HNE can also bind to nuclear hormone receptors, such as peroxisome proliferator-activated receptor delta (PPARδ), promoting the transcription of genes involved in keratinocyte differentiation, such as involucrin and transglutaminase 1, thereby exacerbating epidermal hyperplasia typical of psoriasis [43].

4-HNE also impairs antioxidant defenses by binding to the cysteine residues of key enzymes, including catalase, glutathione peroxidase, and thioredoxin reductase, thus leading to their inactivation and, consequently, to oxidative damage [39,44].

For instance, Zheng et al. demonstrated that 4-HNE can modulate the expression of antioxidant enzymes in mouse keratinocytes through various mechanisms. Specifically, 4-HNE has been shown to induce the nuclear localization of Nrf2, a transcription factor that regulates the expression of antioxidant proteins like heme oxygenase-1 (HO-1). This induction is significantly attenuated in Nrf2-deficient keratinocytes, indicating that 4-HNE’s effects are mediated, at least in part, through the Nrf2 pathway [45].

4-HNE also regulates the Nrf2 pathway by binding to Keap1, its cytosolic inhibitor, promoting its release and allowing Nrf2 nuclear translocation. While Nrf2 typically induces cytoprotective gene expression, its excessive activation in psoriasis skin may paradoxically promote keratinocyte proliferation, thus contributing to disease development [46]. However, the exact interaction between 4-HNE and Nrf2 regulatory proteins has not been fully clarified.

In parallel, 4-HNE activates the NF-κB pathway, a central mediator of psoriatic inflammation. Normally inhibited by IκB, NF-κB becomes activated upon 4-HNE-mediated phosphorylation and the degradation of IκB, leading to the increased expression of pro-inflammatory cytokines [40,46]. 4-HNE also promotes keratinocyte apoptosis by enhancing caspase-3 activity, which contributes to the accelerated epidermal turnover seen in psoriasis. Although direct interaction between 4-HNE and caspase-3 in psoriatic tissue is not fully confirmed, elevated caspase-3 activity and increased 4-HNE-protein adduct formation support its pro-apoptotic role [47,48].

In psoriasis patients, increased levels of 4-oxonenal (4-ONE) protein adducts have been detected in blood cells compared to healthy controls [49].

Like 4-HNE, 4-ONE binds to cysteine, lysine, or histidine residues. In psoriatic lymphocytes, NF-κB and σ 14-3-3 are the most affected proteins. NF-κB, a key inflammatory regulator, undergoes structural modifications that amplify pro-inflammatory signaling, promoting immune cell infiltration and cytokine release, which drive keratinocyte proliferation and lesion progression. Meanwhile, σ 14-3-3, a negative regulator of the cell cycle, becomes impaired, leading to increased keratinocyte proliferation [49].

MDA, another major LPO product, induces immune dysregulation through similar mechanisms. It forms adducts with lysine residues in proteins through Schiff base reactions, leading to MDA–lysine adducts or lysine–MDA–lysine cross-links. Notably, MDA–protein adducts activate protein kinase C (PKC) by binding to it, which triggers IκB phosphorylation. Phosphorylated IκB no longer inhibits NF-κB, consequently leading to its activation [8,50].

Furthermore, MDA–protein adducts stimulate Th17 cells and promote the secretion of pro-inflammatory cytokines such as IL-6, IL-8, and IL-25. MDA levels have been found to be nearly twice compared to healthy controls, confirming its role in psoriasis [40,51]. Although MDA does not directly regulate gene transcription, it influences signaling cascades by altering protein kinase activity. Oxidoreductases, such as NAD(P)H quinone oxidoreductase 1 (NQO1), influence NF-κB activation and cytokine production, while hydrolases, including acyloxyacyl hydrolase and leukotriene-A4 hydrolase, stimulate psoriatic lymphocytes, enhancing inflammatory signaling and interleukin releasing [52,53].

These alterations exacerbate systemic inflammation and promote the migration of activated immune cells to the skin, intensifying keratinocyte hyperproliferation and plaque formation.

**Figure 1:**
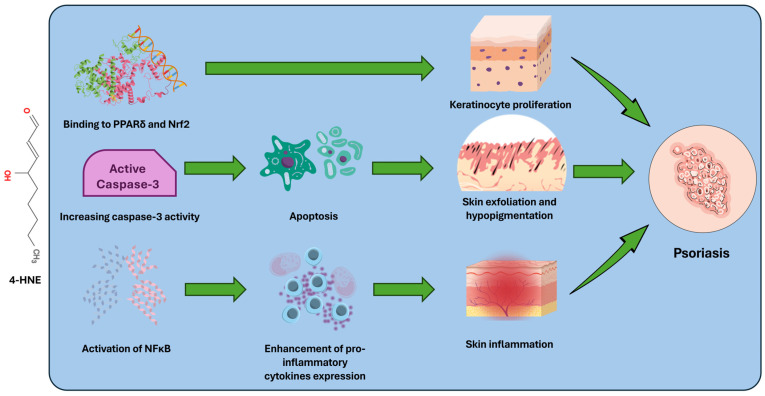
LPO products, particularly 4-HNE, promote the progression of psoriasis through several mechanisms. Evidence suggests that 4-HNE stimulates the proliferation of keratinocytes and their apoptosis, accelerating the process of skin exfoliation. In addition, the same molecule stimulates the production of pro-inflammatory cytokines and thus promotes skin inflammation.

Figure 1 summarizes the major known mechanisms through which 4-HNE and MDA influence the pathogenesis of psoriasis.

### 3.2. Atopic Dermatitis

Atopic dermatitis (AD) is a chronic or chronically relapsing inflammatory skin disease clinically characterized by dry skin and eczema. The disease can affect all ages, with a particular predilection for childhood [54]. The pathogenesis of AD is not yet fully understood. The disease is characterized by a complex interaction between genetic, immunological, and environmental factors [55]. As in many other cutaneous and non-cutaneous pathologies, the role of OS and antioxidant systems is becoming increasingly important. These patients demonstrated a profound alteration in antioxidant/oxidant balance [56]. The skin of atopic patients tries to adapt to this imbalance by implementing antioxidant protection mechanisms. This concept was expressed by Antille et al. [57] in 2002. In the outer layers, the skin barrier mainly uses non-enzymatic antioxidant mechanisms, such as alpha-tocopherol (vitamin E) and ascorbic acid (vitamin C). The outermost layer of the skin, the stratum corneum, has a high lipid density and precisely uses these systems to prevent OS and LPO products from damaging it. Knowing this concept, Antille and his group studied the non-lesional epidermis of 28 patients, including 14 patients with AD and 14 healthy controls. For each sample, the concentrations of alpha-tocopherol in the various layers and the levels of lipid peroxides were evaluated. Patients with AD had higher alpha-tocopherol levels in the stratum corneum compared to the skin of control cases and showed a 25% reduction in the concentration of lipid peroxides. The authors correlated these results to an adaptation mechanism that the skin of atopic patients implements to protect itself from the increased OS that the barrier alteration determines in these patients [57]. The situation is different in the lesional skin of these subjects. Just one year later, Niwa et al. [58] decided to study the oxidative damage of skin proteins in biopsies of lesions of various degrees. They observed a direct correlation between the levels of carbonyl moieties, an indicator of protein oxidation, and the severity of the lesion. Furthermore, using anti-4-HNE antibodies during the immunohistochemical analysis, they observed a more excellent distribution in the layers where these oxidative processes were accentuated, particularly the superficial layers. The authors intuited that 4-HNE could contribute to the oxidative damage of skin proteins [58].

4-HNE is the main product of pollution-induced cutaneous LPO. Ozone, particulate matter, and even cigarette smoke rapidly induce the formation of this aldehyde product. In addition to being a harmful reactive compound, 4-HNE can regulate the affinity of activator protein-1 (AP-1) to DNA, promoting or inhibiting NF-κB activation and, therefore, the inflammatory process. For this reason, air pollution is one of the main risk factors for exacerbating skin lesions in AD patients [59]. Ocular involvement can often occur in atopic patients. It has been demonstrated that OS, particularly LPO and its products, affect the severity of conjunctival inflammation in these patients. Wakamatsu et al. [60] demonstrated a significant positive correlation between the intensity of inflammation and the levels of lipid OS markers such as hexanoyl-lysine and 4-HNE. Several studies in the literature have demonstrated a direct correlation between MDA and AD. In 2013, Sivaranjani et al. [61] evaluated the levels of the main biomarkers of OS and antioxidant systems in a cohort of 25 patients; the results were compared with data from a population of 25 healthy subjects. MDA levels were significantly higher in AD patients compared to healthy controls. On the contrary, antioxidant levels (e.g., catalase, SOD, and vitamins A, C, and E) were lower in the same patients compared to controls. The same results were obtained in 2015 in a case–control study conducted on 130 subjects (65 patients with AD and 65 healthy controls) [62]. Furthermore, some authors have also found a direct correlation between urinary MDA levels and the extent and severity of lesions [63].

Conversely, a 2016 study on children with AD did not show differences in serum MDA levels between AD patients and healthy subjects [64]. The authors justified this result by pointing out the greater regenerative capacity of the redox system in children compared to adults. Recently, a study on AD mouse models focused on oxidative and mitochondrial stress in the epidermis [27]. In these models, the glutathione and catalase systems were reduced, and increased levels of superoxide dismutase 2 and hydrogen peroxide in the mitochondria of keratinocytes were found. In the same cells, a reduction in glutathione peroxidase-4 levels was observed with the generation of high MDA levels in the epidermis. These alterations were not present in Flg−/− models, i.e., mouse models in which the FLG gene (fillagrin) mutation was not present. This result suggests that the FLG gene may contribute to the genesis of OS [27].

### 3.3. Urticaria

Urticaria is a condition characterized by the appearance of wheals, which may or may not be associated with angioedema. The disease varies widely in etiology and the clinical characteristics of skin manifestations. More schematically, today, it is defined as acute or chronic urticaria according to a temporal criterion, which is related to a cut-off of 6 weeks. The etiology is variable: urticaria can be allergic or triggered by chemical or physical factors, but in most cases, there is no identifiable underlying cause, and we speak of chronic spontaneous urticaria (CSU) [65]. The formation of skin lesions is mainly secondary to the activation of mast cells and the release of histamine and other mediators, such as platelet-activating factor (PAF) and cytokines. These factors determine the activation of sensory nerves, vasodilation, and plasma extravasation [66]. Evidence for the role of OS in the pathogenesis of the disease is varied but often conflicting. Several studies have shown that patients with CSU had reduced levels of antioxidants and increased oxidation products [67].

On the other hand, as early as 2007, the first piece of evidence questioning the role of SO and LPO products in urticaria was found. Kasperska-Zajac and her group studied antioxidant systems in the plasma and red blood cells of a group of patients with CSU (including 14 female patients with positive autologous serum test, 31 negative patients, and 19 healthy patients) [68]. Furthermore, MDA levels were evaluated as a biomarker of LPO. No significant differences were found between the three groups of patients, demonstrating the low importance of the oxidant/antioxidant system in the pathogenesis of the disease. One year later, the same author re-evaluated the activity of antioxidant enzymes, including Copper Zinc Superoxide Dismutase (Cu/ZnSOD), GSH-Px, and catalase, and the levels of MDA in plasma and erythrocytes of 12 women with non-steroidal anti-inflammatory drug (NSAID)-induced urticaria and 19 healthy controls. The results confirmed that there were no differences between the two groups and that even in acute urticaria from NSAID, OS and LPO did not play a role [69].

A few years later, Sagdic et al. [70] identified a potential alteration in the antioxidant system of patients with CSU. Their study on 25 patients with urticaria and 36 healthy controls showed a statistically significant decrease in the Cu/ZnSOD activity of erythrocytes compared to the controls. However, the levels of MDA of erythrocytes in patients with CSU were not different from those of healthy subjects.

Rajappa et al. [71] evaluated the platelet oxidant/antioxidant system in 45 patients with CSU compared to 45 healthy controls matched for age and sex. The study demonstrated that patients with CSU had significantly decreased platelet SOD and GPx levels and significantly elevated platelet MDA levels compared to healthy controls. These results indicate a possible implication of platelet OS in patients with CSU. In 2012, a study highlighted the potential role of OS in immunopathogenesis in the clinical spectrum of cutaneous drug reactions, including acute allergic urticaria. The mean MDA levels during the allergic reaction were significantly increased compared to baseline and healthy subjects. Furthermore, a positive correlation could be observed between MDA levels and the positivity rate of the drug response to leukocyte migration inhibition, a test to confirm the immunological bases of the reaction [72].

Subsequent studies have also confirmed the increased oxidative load and the deficit of antioxidant systems in acute urticaria forms, particularly correlating with increased serum MDA levels [73].

### 3.4. Vitiligo

Vitiligo is a chronic acquired skin disorder characterized by the selective loss of melanocytes, leading to patchy depigmentation. Although many theories have been proposed, it is now recognized that vitiligo has a multifactorial pathogenesis, including gene polymorphisms, autoimmune responses, and OS [74,75]. Vitiligo is characterized by an imbalance between the production of ROS and the capacity of the antioxidant defense system, leading to increased oxidative damage within melanocytes. This redox imbalance is particularly detrimental to these cells due to their high metabolic activity and susceptibility to oxidative insults [76]. Melanin synthesis itself is linked to ROS generation, as melanin absorbs UV light, contributing to OS. Elevated ROS levels in vitiliginous skin arise from environmental triggers (e.g., UV radiation and chemical exposure), impaired antioxidant enzyme activity, and mitochondrial dysfunction [77]. ROS accumulation leads to LPO, protein oxidation, and DNA damage, ultimately impairing melanocyte survival [78]. Furthermore, OS disrupts key cellular pathways, including the Nrf2 antioxidant response pathway and the MAPK signaling cascade, reducing the cell’s ability to counteract oxidative damage [79].

Beyond direct cytotoxic effects, OS plays a pivotal role in initiating immune activation in vitiligo. Damaged melanocytes release danger-associated molecular patterns (DAMPs), including heat shock proteins (HSPs) and oxidized lipids, which activate innate immune responses and promote the recruitment of autoreactive T cells, mediated by the release of pro-inflammatory cytokines, including IL-15, IL-17, and IL-1beta [80,81].

Moreover, OS is implicated in the dysfunction of melanocyte adhesion, caused by the ROS-mediated disruption of E-cadherin expression, thus contributing to melanocyte detachment and loss [82].

To corroborate the role of OS in vitiligo, several studies have reported a correlation between the accumulation of radicals and ROS and alterations in both blood and epidermal components of vitiliginous skin [83,84].

Reactive aldehydes, including MDA and 4-HNE, are key markers and mediators of OS-induced cellular damage, whose accumulation contributes to melanocyte dysfunction and death, ultimately leading to depigmentation [85].

MDA forms adducts with proteins and nucleic acids, altering their function and triggering immunogenic responses. Its ability to modify cellular macromolecules contributes to melanocyte apoptosis, further perpetuating the cycle of oxidative damage [76]. Similarly, 4-HNE interacts with proteins, phospholipids, and DNA, impairing cellular function and modulating pathways involved in survival, apoptosis, and inflammation [86].

These aldehydes also activate immune responses by functioning as DAMPs, stimulating dendritic cells and inducing pro-inflammatory cytokines such as IL-6, IL-1β, and TNF-α, contributing to vitiligo’s autoimmune component [87]. Their interactions with key intracellular pathways, such as the nuclear factor Nrf2 pathway and MAPK signaling cascade, further amplify melanocyte vulnerability [88]. On this topic, significantly higher levels of LPO products, including MDA, and significantly lower levels of serum SOD activity were found in patients with generalized vitiligo compared to healthy controls, highlighting the role of OS and LPO in vitiligo pathogenesis [89,90]. Other authors studied the levels of MDA, catalase, GSH-Px, and SOD in the tissues of 10 patients with active vitiligo, 10 patients with stable vitiligo, and 20 matched healthy controls. The results revealed that the levels of SOD, GSH-Px, and MDA in the tissues were significantly increased in patients with active vitiligo compared with those with stable vitiligo and matched controls. This evidence suggests that OS is not only correlated with disease onset but also with disease activity [91].

Given this pathogenic framework, antioxidant therapies may help counteract oxidative damage, restore redox homeostasis, and potentially slow disease progression. Emerging evidence suggests that compounds with antioxidant properties, such as vitamins C and E, polyphenols, and enzymatic cofactors, may help protect melanocytes from oxidative injury, supporting their inclusion as an adjunctive approach in vitiligo treatment [75].

**Figure 2:**
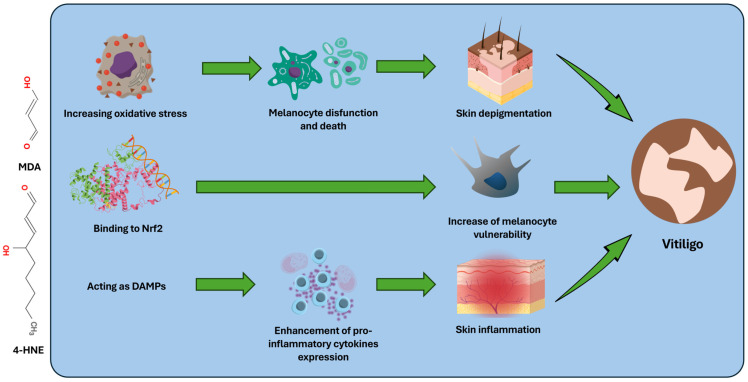
MDA and 4_HNE enhance oxidative stress, promote melanocyte apoptosis, and favor the skin depigmentation processes underlying the disease. Furthermore, even in the pathogenesis of vitiligo, these molecules promote inflammatory processes by acting as DAMPs. Finally, the bond with Nrf2 increases melanocyte vulnerability and promotes cell death.

Figure 2 summarizes the main known mechanisms through which 4-HNE and MLD influence the pathogenesis of vitiligo.

### 3.5. Alopecia Areata

Alopecia areata (AA) is an acquired, inflammatory, and autoimmune condition characterized by patches of non-scarring hair loss, commonly affecting the scalp or any other part of the hair-bearing skin [92].

The collapse of hair immune privilege is one of the most widely accepted pathogenetic theories. However, the exact onset mechanisms are not fully elucidated. Various factors are supposed to contribute to disease development, including genetic predisposition, allergies, microbiota, and psychological stress. Notably, OS and increased ROS production from perifollicular inflammatory cells are believed to be linked to AA, triggering the breakdown of hair follicle immune privilege [93].

LPO represents the hallmark of OS, which results after the exposition of cell membrane lipids to ROS [94]. The peroxidation of membrane lipids generates reactive aldehydes, which form adducts with proteins, phospholipids, and DNA. These LPO products modulate signaling pathways involved in apoptosis, immune activation, and inflammatory responses, exacerbating follicular immune privilege collapse and facilitating T-cell-mediated attacks on hair follicles [93]. LPO products induce oxidative damage to follicular structures, thus blocking the hair growth cycle and promoting an inflammatory microenvironment, which finally leads to hair loss. To corroborate this hypothesis, several studies evaluated the levels of MDA in serum and scalp tissues of patients with AA, highlighting higher levels of MDA compared to controls [94,95] and a correlation between disease duration and severity with MDA levels [96]. This oxidative imbalance was further evidenced by altered activities of antioxidant enzymes, including SOD and GSH-Px. Specifically, some studies reported decreased SOD activity in the serum of AA patients [97], while others found increased SOD and GSH-Px levels in scalp tissues, thus suggesting a complex and localized response to OS [98]. Additionally, a higher OS index and total oxidant capacity values were correlated with severe manifestations of AA, further underscoring the potential role of LPO in disease progression [95].

### 3.6. Pemphigus

Pemphigus is a term used to describe a family of rare autoimmune dermatoses characterized by acantholysis in which the loss of cell–cell adhesion is responsible for the formation of blisters and mucocutaneous erosions [99]. Several subtypes of pemphigus include pemphigus vulgaris (PV), pemphigus foliaceous (PF), IgA pemphigus, and paraneoplastic pemphigus. The disease pathogenesis is based on the presence of autoantibodies directed against proteins on the surface of keratinocytes, called desmogleins. These transmembrane glycoproteins associated with desmosomes confer cell–cell adhesion within the epidermis [99].

As in many other skin diseases, the role of the oxidant/antioxidant system could also be relevant in the pathogenesis of pemphigus. Indeed, the study of OS indices, total antioxidant capacity, and lipid hydroperoxide levels in PV patients confirms this hypothesis. Yesilova and his group showed that the OS indices and lipid hydroperoxide levels in PV patients were higher than those in healthy subjects. However, they did not find a significant reduction in total antioxidant capacity [100].

The first studies regarding LPO in PV patients were conducted by Naziroğlu et al. [101] in 2003. This group evaluated the antioxidant vitamins, glutathione, GSH-Px, catalase, and Malate dehydrogenase (MDH) levels in 18 non-smoking PV patients and compared them with healthy controls. Plasma and red blood cells from PV patients showed significantly higher levels of MDH and reduced plasma levels of antioxidant vitamins compared with healthy controls. Furthermore, the activity levels of antioxidant enzymes were significantly reduced in PV patients, although there were no differences in GSH-Px levels [101]. Subjects with PV show elevated MDH levels, which correlate positively with anti-desmoglein antibody levels [102]. However, in the early stages of the disease, the role of LPO may be limited. Javanbakht et al. [103] studied the antioxidant and oxidant activities in patients with newly diagnosed PV. In particular, they observed how the activity of the antioxidant system, measured by catalase, GPx, and SOD, was increased in these patients. However, the plasma MDH levels did not differ between the PV group and the control cases at diagnosis. LPO also plays a key role in the pathogenesis of PF. A study on a Tunisian population composed of 13 PF patients and seven healthy subjects evaluated MDH levels in biopsies of lesional, perilesional, and healthy skin [104]. The MDA levels in the skin biopsies were significantly higher in the samples from PF patients. The results were later confirmed by a new study conducted in 2018 on a Peruvian population. In this case, the plasma MDH levels were evaluated in patients with active disease and healthy subjects with anti-desmoglein1 (anti-dsg1) antibodies. Interestingly, MDH levels, compared to healthy subjects, were higher in patients with PF and healthy subjects with anti-dsg1 [105]. Furthermore, the levels of these antibodies are positively correlated with MDH levels in patients with PF [102].

Currently, there is no evidence regarding the role of LPO in the pathogenesis of IgA pemphigus and paraneoplastic pemphigus.

### 3.7. Melanoma

Melanoma is an aggressive skin cancer originating from the malignant transformation of melanocytes. Its pathogenesis is influenced by familiar history, genetic susceptibility, and environmental and metabolic factors. Among these, OS, characterized by an imbalanced release of ROS, is closely linked to melanoma development. It induces DNA damage, promotes oncogene activation, and modulates signaling pathways, thus enhancing tumor growth and metastasis [106,107]. LPO, the ROS-driven oxidation of PUFAs, leads to the formation of bioactive aldehydes like 4-HNE and MDA, playing a key role in OS processes. In melanoma, LPO products can exert both pro- and anti-tumorigenic effects depending on their concentration, cellular context, and tumor stage [108].

Significantly elevated MDA levels have been found in melanoma tissues compared to control samples [109]. Several studies have also reported increased MDA concentrations in the plasma of melanoma-bearing mice and human patients [110]. Notably, serum MDA levels were elevated at all melanoma stages, with the highest levels being observed in stage IV patients, thus suggesting a direct correlation between tumor stage and LPO levels [111].

While MDA is widely recognized for its mutagenic properties, 4-HNE has more complex biological functions, acting as both a signaling molecule and a cytotoxic agent. In a cohort including simple nevi, dysplastic nevi, primary malignant melanomas, and metastatic lesions, immunohistochemical analyses revealed significantly increased 4-HNE levels in dysplastic nevi compared to benign nevi while remaining stable in cutaneous malignant melanoma and markedly reduced in metastases. These findings suggest that 4-HNE may play a role in the early stages of melanoma tumorigenesis, while its loss in metastases may be associated with the increased proliferative activity of metastatic cells [112]. Indeed, at micromolar concentrations comparable to those found in human plasma and tissues, 4-HNE has demonstrated antiproliferative, proapoptotic, antiangiogenic, and prodifferentiative effects on various tumor cells in vitro. These effects are mediated by the modulation of key oncogenes, tumor suppressor genes, transcription factors, apoptotic regulators, and microRNAs [113,114,115]. Pioneering research established that 4-HNE negatively regulates tumor cell growth by modulating the expression of oncogenes such as c-myc, c-myb, and c-fos, either independently or in conjunction with serum growth factors [116,117,118,119].

On this topic, 4-HNE anticancer activity has been extensively demonstrated in melanoma cells. Early studies showed that HNE treatment inhibited B16-F10 melanoma cell proliferation both in vitro and in vivo. Furthermore, the exposition of pigmented (B16-F10) and amelanotic (B16BL6) murine melanoma cells to cytotoxic 4-HNE concentrations conferred resistance to OS in surviving cells, possibly due to the formation of a bioactive conjugate with an extracellular peptide or protein present in serum [120]. This conjugate, observed exclusively in the presence of serum, was proposed to mediate HNE’s tumor-suppressive effects [121].

**Figure 3:**
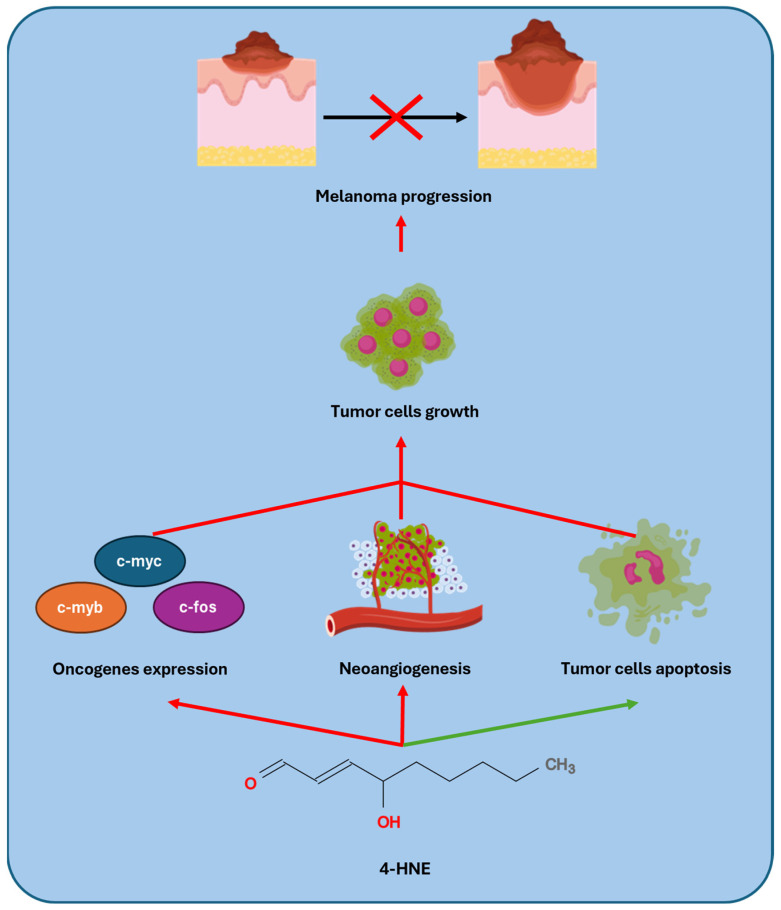
A high dose of 4-HNE causes the suppression (red arrow) of oncogenes and neoangiogenesis in tumor cells, reducing the risk of metastasis. Furthermore, the cytotoxic action of this molecule promotes apoptosis (green arrow). Overall, tumor growth is reduced, and the progression of the neoplastic lesion is slowed.

Subsequently, to exploit the anti-tumor properties of 4-HNE in vivo, the use of nanovehicles to enhance the delivery of this highly reactive and poorly soluble aldehyde has been explored. Specifically, β-cyclodextrin-poly conjugates and β-cyclodextrin-based lipid nanocapsules loaded with 4-HNE have been found to amplify its anticancer effects in melanoma cells, thus highlighting their potential for clinical use [122,123]. Figure 3 illustrates the anti-tumor proprieties of 4-HNE.

Furthermore, there is strong evidence about the role of ferroptosis in regulating tumor cell death in the literature. Ferroptosis, a cell death mechanism driven by accumulated iron-dependent lipid ROS, is not only a process capable of blocking cell growth but also increases sensitivity to chemotherapeutic drugs [124]. Therefore, although the role of ferroptosis in tumorigenesis is still unclear, it is now known that acting positively on ferroptosis can be a valid therapeutic strategy in therapy-resistant tumors. In a recent review, Ta et al. [125] analyzed the role of ferroptosis in melanoma, especially how the regulation of the process can be a promising therapeutic strategy. Melanoma tumor cells are rich in PUFAs and have reduced glutathione activity. Therefore, they are highly susceptible to this type of cell death. Numerous small molecules and nano-materials capable of inducing the ferroptosis pathway and blocking tumor growth are being studied, also enhancing the effect of immunotherapy or the action of BRAF inhibitors. The current limit is to identify tumor biomarkers that can indicate the sensitivity of cells to this type of therapy [125].

## 4. Discussion

LPO is the biochemical process in which free radicals and ROS oxidize PUFAs within cellular membranes, thus generating reactive aldehydes, including MDA and 4-HNE, that serve as mediators and markers of oxidative damage [8,9]. LPO plays a dual role in the skin, an organ continuously exposed to both endogenous metabolic byproducts and exogenous insults. Under physiological conditions, controlled LPO participates in redox signaling and barrier homeostasis by modulating the composition of epidermal lipids, including ceramides and free fatty acids [23]. However, when antioxidant defenses are overwhelmed by oxidant insults, as happens in several cutaneous conditions, excessive LPO triggers inflammatory cascades, which lead to tissue damage.

In psoriasis, increased levels of MDA and 4-HNE have been consistently observed in lesional skin, correlating with disease severity and PASI score. These aldehydes modify critical proteins via adduct formation, alter intracellular signaling, and promote the activation of NF-κB and other pro-inflammatory pathways [34,35], which exacerbate local inflammation and contribute to the systemic oxidative burden observed in psoriatic patients [36].

Natural antioxidants, such as cannabidiol (CBD), show therapeutic promise for psoriasis due to their antioxidant and anti-inflammatory effects. CBD protects skin cells from UV-induced oxidative stress and reduces 4-HNE-protein adduct formation by up to 80% [126,127]. Topical CBD has been associated with reduced keratinocyte proliferation and the improvement in psoriatic lesions, including decreased erythema and scaling [128]. Other natural compounds with antioxidant properties, such as aloe vera, quercetin, curcumin, resveratrol, baicalein, and bergamot essential oil, have been investigated for psoriasis management. Aloe vera modulates immune responses and inhibits NF-κB signaling, while quercetin reduces pro-inflammatory cytokines (TNF-α, IL-6, and IL-17). Curcumin suppresses keratinocyte proliferation and inflammatory mediators like IL-1β and IL-22. Resveratrol promotes keratinocyte apoptosis and reduces IL-17 levels, and baicalein and bergamot essential oil exert antioxidant and anti-inflammatory effects by modulating the IL-17 pathway and regulating keratinocyte activity. These compounds may serve as adjuncts or alternatives to conventional treatments with fewer side effects [129].

Moving to AD, it exemplifies another condition where LPO plays a crucial role. In non-lesional AD skin, an adaptive increase in non-enzymatic antioxidants, such as α-tocopherol, is correlated with a reduction in lipid peroxides, suggesting a compensatory mechanism aimed at mitigating oxidative damage [57]. However, during active disease phases, lesional skin exhibits marked oxidative damage with higher 4-HNE levels, highlighting that antioxidant defenses are overwhelmed [58].

In urticaria, the role of OS and LPO imbalance remains unclear, with limited and contradictory evidence available [69,72]. Conversely, in PV, increased OS markers, including MDA, suggest that LPO may contribute to acantholysis and blister formation [101].

In vitiligo, elevated ROS levels and consequent LPO-derived products such as MDA and 4-HNE contribute to melanocyte cytotoxicity and function as DAMPs, triggering innate immune responses and promoting autoreactive T-cell recruitment, which further drives melanocyte destruction [85,86].

**Figure 4:**
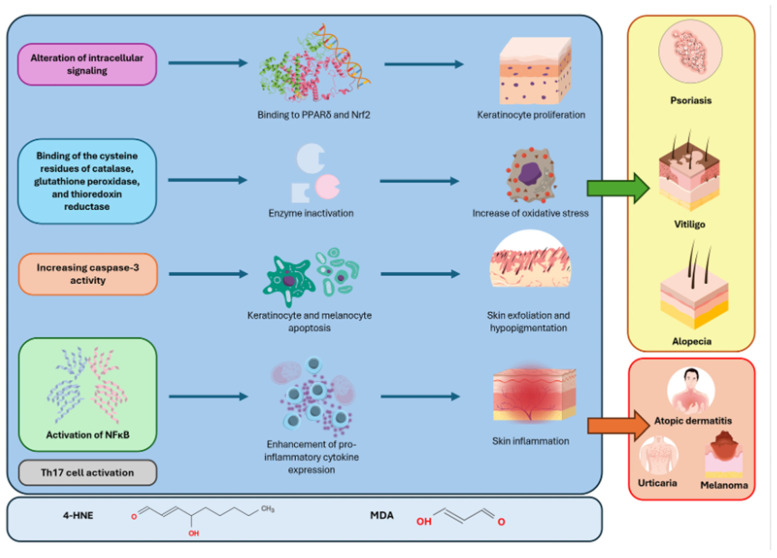
The roles of MDA and 4-HNE in skin diseases. The green arrow indicates the diseases for which there is evidence of a pathogenetic role of MDA and/or 4-HNE. The red arrow indicates the diseases in which the levels of LPO biomarkers are increased but for which there is no evidence of a pathogenetic role.

AA also exhibits LPO involvement, with increased MDA levels in both serum and scalp tissue correlating with disease severity and duration [94,98]. The peroxidation of membrane lipids in hair follicles triggers apoptotic signaling cascades and promotes a pro-inflammatory microenvironment, thereby enhancing T-cell-mediated attacks on follicular structures. Figure 4 summarizes the role of LPO aldehyde products in the pathogenesis of the skin diseases described.

Table 1 lists and summarizes the evidence regarding the possible pathogenic role of LPO products in skin diseases.

Moving to skin cancer, melanoma presents a complex scenario where LPO exerts both pro-tumorigenic and anti-tumorigenic effects. While elevated MDA levels in melanoma tissues underscore the role of OS in tumoral progression, on the other hand, the cytotoxic properties of 4-HNE at controlled concentrations have been shown to inhibit cancer cell proliferation and induce apoptosis [109,120]. Emerging research suggests that inducing LPO selectively targets melanoma cells via ferroptosis, an iron-dependent and non-apoptotic form of programmed cell death characterized by the accumulation of lipid peroxides and ROS, which finally leads to membrane damage and cell death [130]. Melanoma cells, particularly those with high metabolic plasticity, may be selectively vulnerable to LPO-driven ferroptosis due to their dependence on specific antioxidant defense systems [131,132]. One key mechanism through which LPO exerts its cytotoxic effects in melanoma involves glutathione metabolism disruption and glutathione peroxidase 4 (GPX4) inhibition. GPX4 reduces lipid hydroperoxides to non-toxic lipid alcohols, thereby preventing oxidative membrane damage. The pharmacological inhibition of GPX4 or the depletion of its cofactor, glutathione, leads to the accumulation of oxidized lipid species, ultimately triggering ferroptosis [86]. This suggests that therapies targeting GPX4 could be particularly effective in melanomas resistant to apoptosis-inducing therapies, such as BRAF and MEK inhibitors [125].

Iron metabolism also contributes to LPO-induced ferroptosis in melanoma [133]. Iron overload enhances LPO through Fenton chemistry, whereby free iron catalyzes hydrogen peroxide conversion into highly reactive hydroxyl radicals, promoting oxidative damage to cellular membranes. Melanoma cells often exhibit dysregulated iron homeostasis, with increased iron import and storage, making them more susceptible to OS-induced cell death [134]. Ferroptosis-inducing agents, which enhance intracellular iron levels, may serve as a future therapeutic strategy in melanoma management [125].

In addition to these intrinsic mechanisms, melanoma cells exhibit antioxidant defense systems which maintain intracellular glutathione levels. Inhibiting this system, either pharmacologically or genetically, leads to glutathione depletion, promoting LPO and cell death via ferroptosis [135]. Furthermore, the Nrf2 pathway, a key regulator of OS responses, is often upregulated in melanoma, conferring resistance to ferroptosis. Suppressing Nrf2 activity could therefore enhance the efficacy of ferroptosis-inducing strategies [134].

From a therapeutic standpoint, targeting LPO to induce ferroptosis may represent a promising approach for melanoma treatment, particularly in cases that have developed resistance to standard therapies, which represent a true challenge for physicians. Combining ferroptosis-inducing agents with already available therapies, such as immune checkpoint inhibitors and targeted kinase inhibitors, may provide synergistic effects and improve patients’ clinical outcomes and survival [134]. Future research should focus on optimizing ferroptosis-inducing strategies while minimizing potential toxicities to normal tissues, ultimately advancing this novel approach toward clinical application [125,134].

Another emerging adjuvant treatment for melanoma is represented by photodynamic therapy (PDT), which combines a photosensitizing agent, light exposure, and oxygen [136,137]. Upon activation by specific wavelengths of light, the photosensitizer generates ROS, leading to OS and subsequent cellular damage [138]. Among the oxidative processes induced by PDT, LPO and MDA accumulation disrupt cellular homeostasis and trigger apoptotic pathways [139]. Specific photosensitizers, such as 4-hydroxyphenyl porphyrin (THOPP), which preferentially accumulate in mitochondria, trigger oxidative damage and enhance apoptotic signaling via caspase-3 activation [140].

LPO and MDA accumulation play critical roles in PDT-induced cytotoxicity in melanoma. Strategies that enhance oxidative damage while minimizing antioxidant defense activation may improve PDT efficacy, offering a viable therapeutic approach for treatment-resistant melanomas.

Finally, in recent years, the use of artificial intelligence (AI) and machine learning (ML) in the medical field has become widespread. ML has been widely applied in the biochemical field and in studying redox systems and OS. In particular, in recent years, several authors have focused their attention on developing models for assessing, categorizing, and predicting OS. It seems that supervised ML models, such as those based on logistic regression, decision trees, and neural networks, could optimize the process of assessment and quantification of OS [141]. For example, models have been developed that are useful for studying the DNA damage induced by OS and, therefore, the potential underlying mechanisms [142]. The application of ML is also proving useful in the study of LPO in various diseases, allowing for the creation of models capable of providing an early diagnosis of the disease, helping the clinician in more complex diagnoses, or even in identifying groups of genes involved in the LPO process and in the pathogenesis of the disease [143,144].

## 5. Conclusions

LPO and its products, particularly MDA and 4-HNE, are essential in the pathogenesis of several pathologies. While in conditions of balance between oxidant and antioxidant systems, LPO derivatives can play a protective role in the skin, at high concentrations, they can play an active role in the pathogenesis of inflammatory and non-inflammatory skin diseases. In the literature, there is evidence about the roles of MDA and 4-HNE in the inflammatory process, which occurs in diseases such as psoriasis and vitiligo. In particular, the aldehyde derivatives of LPO promote the production of pro-inflammatory cytokines and the apoptosis of keratinocytes and melanocytes, and they inhibit antioxidant enzymes. These same products promote and perpetuate oxidative stress. The exact mechanisms by which LPO products may influence the pathogenesis of other skin diseases, such as AD, urticaria, and pemphigus, are poorly understood, and the literature only contains hypotheses. It is clear that there is a correlation between the pathology and the levels of LPO markers, but their role in the pathogenesis of these diseases is not clear. The therapeutic role of LPO in treating neoplastic diseases is noteworthy. By exploiting the toxic effects of 4-HNE, it is possible to counteract the proliferation of neoplastic cells. This offers new perspectives for treatment not only in melanoma but also in other tumors. It is therefore necessary to continue studying the LPO process and the role of its products in the course of the main skin diseases to determine not only disease or severity markers but also targeted therapeutic approaches. Combining a targeted therapy against LPO and other conventional therapies or exploiting LPO itself could lead to a turning point in treating various inflammatory or neoplastic skin diseases, minimizing side effects, and increasing their efficacy.

Finally, applying new AI technologies and ML models to study OS and LPO will undoubtedly lead to deeper knowledge about their role in many pathologies, including skin diseases. These tools will also provide efficient diagnostic and prognostic algorithms for a better therapeutic approach.

## Figures and Tables

**Table 1:** The main evidence in the literature regarding the role of LPO products in the pathogenesis of skin diseases.

Author, Year	Skin Disease	Molecules	Results
Gęgotek et al. [39], 2019	Psoriasis	4-HNE	The proteomic approach showed an increase in the level of 4-HNE protein adducts. Among inactivated proteins, many are involved in the antioxidant system.
Wójcik et al. [40], 2019	Psoriasis	4-HNE	Patients with psoriasis show elevated levels of 4-HNE-adducts and an alteration in lipid metabolism with an enhancement in mediators that modulate the immune system in mononuclear cells.
Blunder et al. [43], 2021	Psoriasis, AD	General LPO products	LPO products bind to PPARδ, promoting keratinocyte differentiation and exacerbating epidermal exfoliation.
Yang et al. [46], 2017	Psoriasis	4-HNE	4-HNE regulates Nrf2 by binding to cysteine residues of its inhibitor, Keap1. The excessive activation of Nrf2 promotes keratinocyte proliferation, thus contributing to the development of skin lesions.
Niwa et al. [58], 2003	AD	4-HNE	In an immunohistochemical analysis of skin samples from AD subjects, anti-4-HNE antibodies are more intensely distributed on the superficial layers, areas where OS is more expressed. These findings suggest that 4-HNE increases OS in patients with AD.
Wang et al. [87], 2022	Vitiligo	LPO	OS and LPO products promote the release of DAMPs from keratinocytes and melanocytes in the skin, inducing immune responses.
Koca et al. [89], 2004	Vitiligo	MDA	Serum MDA levels in vitiligo patients are significantly increased compared to healthy controls. This leads to increased OS and damage to the melanocyte cell membrane.
